# Effects of interacting networks of cardiovascular risk genes on the risk of type 2 diabetes mellitus (the CODAM study)

**DOI:** 10.1186/1471-2350-9-36

**Published:** 2008-04-24

**Authors:** Marleen MJ van Greevenbroek, Jian Zhang, Carla JH van der Kallen, Paul MH Schiffers, Edith JM Feskens, Tjerk WA de Bruin

**Affiliations:** 1 Laboratory for Metabolism and Vascular Medicine, Department of Internal Medicine/Cardiovascular Research Institute (CARIM), Maastricht University, Maastricht, The Netherlands.; 2 Institute of Mathematics, Statistics and Actuarial Sciences, University of Kent, Canterbury, UK.; 3 Department of Pharmacology and Toxicology, Maastricht University, Maastricht, The Netherlands.; 4 Division of Human Nutrition, Section Nutrition and Epidemiology, Wageningen University, Wageningen, The Netherlands.

## Abstract

**Background:**

Genetic dissection of complex diseases requires innovative approaches for identification of disease-predisposing genes. A well-known example of a human complex disease with a strong genetic component is Type 2 Diabetes Mellitus (T2DM).

**Methods:**

We genotyped normal-glucose-tolerant subjects (NGT; n = 54), subjects with an impaired glucose metabolism (IGM; n = 111) and T2DM (n = 142) subjects, in an assay (designed by Roche Molecular Systems) for detection of 68 polymorphisms in 36 cardiovascular risk genes. Using the single-locus logistic regression and the so-called haplotype entropy, we explored the possibility that (1) common pathways underlie development of T2DM and cardiovascular disease -which would imply enrichment of cardiovascular risk polymorphisms in "pre-diabetic" (IGM) and diabetic (T2DM) populations- and (2) that gene-gene interactions are relevant for the effects of risk polymorphisms.

**Results:**

In single-locus analyses, we showed suggestive association with disturbed glucose metabolism (i.e. subjects who were either IGM or had T2DM), or with T2DM only. Moreover, in the haplotype entropy analysis, we identified a total of 14 pairs of polymorphisms (with a false discovery rate of 0.125) that may confer risk of disturbed glucose metabolism, or T2DM only, as members of interacting networks of genes. We substantiated gene-gene interactions by showing that these interacting networks can indeed identify potential "disease-predisposing allele-combinations".

**Conclusion:**

Gene-gene interactions of cardiovascular risk polymorphisms can be detected in prediabetes and T2DM, supporting the hypothesis that common pathways may underlie development of T2DM and cardiovascular disease. Thus, a specific set of risk polymorphisms, when simultaneously present, increases the risk of disease and hence is indeed relevant in the transfer of risk.

## Background

Genetic strategies for dissection of complex diseases require innovative approaches for identification of disease-predisposing genes or -combinations of polymorphisms. The development and progression of complex diseases involve interplay of genetic and environmental factors. This implies involvement of several susceptibility genes in the development of a complex disease, and an interaction between these genes and the environment, e.g. lifestyle habits [1]. Not only gene-environment interactions, but also gene-gene interactions may be highly relevant for development of a complex disease.

Type 2 Diabetes Mellitus (T2DM) is an example of a complex, multigenic disorder with a high prevalence in man [2,3]. A large number of candidate susceptibility genes and loci for T2DM have been proposed and some of those have been replicated in more than one study population using linkage analysis (reviewed by Stern [4]).

It becomes increasingly clear that simple assessment of the contribution of individual genes to T2DM susceptibility via analysis of the individual effects of common polymorphisms may not be sufficient for identification of the processes and genes involved in T2DM. Indeed, genetic interactions in T2DM have been described for some pairs of genes or SNPs [5-7]. Therefore, these polymorphisms should be studied both individually and as members of genetic networks. To address complex relations between potential susceptibility genes, Zhang et al. [8] developed a method for detecting complex haplotype interactions (or allele coupling) among a set of polymorphisms, which may influence the susceptibility to a complex disease. The advantage of this method over single-locus analyses lies in the fact that it allows for identification of the combined presence of specific (functional) SNPs in disease. This approach has shown to be very effective for the analysis of gene-gene interactions, where genetic polymorphisms do not reside on the same chromosome, i.e. are evidently physically unlinked, but interact with each-other to contribute to the risk of complex disease [9]. The rationale behind this approach is that simultaneous presence of particular gene variations, even when physically unlinked, may predispose to disease because some variations may affect the "local environment" (e.g. insulin resistance or glucose homeostasis) in a way that affects the consequences of other variations (e.g. reduced beta-cell function).

T2DM is known to be associated with increased risk of cardiovascular disease and we hypothesize that the increased cardiovascular risk in T2DM implies common metabolic pathways for development of T2DM and cardiovascular disease and, hence, that "pre-diabetic" (IGM) and diabetic (T2DM) populations will be enriched in cardiovascular risk polymorphisms. This motivated us to use an assay for candidate markers of cardiovascular risk designed by Roche Molecular Systems [10] for our current analyses.

For this study we determined a set of known cardiovascular risk polymorphisms in the CODAM (Cohort Study on Diabetes and Atherosclerosis Maastricht) population [11]. We used both single locus-based logistic regression and the method developed by Zhang et al. [8] to explore the possibility that complex haplotype interactions (or allele coupling) in this set of cardiovascular risk polymorphisms can be implicated in risk of glucose intolerance or T2DM.

## Methods

### Description of subjects

The Cohort study of Diabetes and Atherosclerosis Maastricht (CODAM) is a prospective population-based cohort study in The Netherlands [10]. Inclusion criteria were: age 40–70 years and Caucasian descent (i.e. four Caucasian grandparents), and in addition either a body mass index (BMI)>25 kg/m2, and/or a positive family history for T2DM, and/or a history of gestational diabetes, and/or the use of antihypertensive medication, and/or a postprandial blood glucose larger than 6.0 mmol/l and/or glucosuria. All subjects were genetically independent (i.e. unrelated) and the NGT, IGM and T2DM phenotype was assigned based on the results of an oral glucose tolerance test. IGM subjects were either impaired glucose tolerant or had impaired fasting glucose levels. A majority of the subjects (>85%) were unaware of their glucose tolerance status prior to inclusion in this cohort. The study was approved by the local Medical Ethical Committee of the Maastricht University/Maastricht University Hospital and all subjects gave written informed consent. All subjects with disturbed glucose metabolism (i.e. subjects who were IGM or who had T2DM) and a random sample of the control (NGT) individuals were included in the current study. A summary of the characteristics of these subjects is provided in Table 1.

**Table 1:** Basic characteristics of the subjects.

	NGT	IGT	T2DM
Nô	54	111	141
male subjects (%)	57 %	58 %	66 %
Age (Yrs)*	58.8 ± 7.9	59.5 ± 6.6	60.8 ± 6.2
BMI (kg/m2)*	27.6 ± 4.1	28.8 ± 4.0	30.4 ± 4.6
Mean arterial pressure (mm Hg)*	113.3 ± 13.6	120.9 ± 15.4	124.9 ± 14.8
Fasting Glucose (mM)*	5.3 ± 0.4	5.9 ± 0.5	8.0 ± 1.8
Fasting Insulin (μU/L)*	8.7 ± 4.9	11.0 ± 6.2	14.3 ± 8.9
HDL-cholesterol (mM)*	1.2 ± 0.3	1.1 ± 0.3	1.0 ± 0.3
Triglycerides (mM)*	1.4 ± 0.7	1.7 ± 0.8	2.1 ± 1.5
Coronary heart disease (%)	17 %	13 %	13 %

* mean ± standard deviation.

### Genotyping

Research assays for cardiovascular disease genetics designed by Roche Molecular Systems [11] were used to genotype 68 SNPs in genes in pathways of lipid and homocystein metabolism, regulation of blood pressure and coagulation, inflammation, cellular adhesion, and matrix integrity (listed in Table 2). DNA samples of the subjects included in the study were genotyped by using the polymerase chain reaction (PCR). This led to 309 genotypes of 68 loci that can each be assigned to one of the following three groups: a NGT (control) group (n = 54), a glucose intolerant group (n = 111 IGM subjects), and a T2DM group (n = 142 patients). Each genotype can be divided into 16 blocks according to their chromosome identities. See Table 2. A cut-of value of ≥ 21% for "heavy missing" was selected on the basis of experience [8]. We decided not to include the APOE(Arg158Cys) locus in the logistic regression analyses because it had a missing rate of 41.7% (≥ 21%). All other loci had less than 10% missing.

**Table 2:** Polymorphisms used in genotyping assay.

Block and SNP (symbol)	Location	Gene name (OMIM #)*	Missing rate (%)
1:			
C677T (V1)	1p36.3	MTHFR (MIM 607093)	0.32
Met235Thr (V2)	1q42	AGT (MIM 106150)	0.32
G664A (V3)	1p36.2	NPPA (MIM 108780)	0.32
T2238C (V4)	1p36.2	NPPA	0.32
Arg506Gln (V5)	1q23	F5 (MIM 227400)	0
Ser128Arg (V6)	1q23	SELE (MIM 131210)	0.32
Leu554Phe (V7)	1q23	SELE	0.32
2:			
Thr71Ile (V8)	2p24	APOB (MIM 107730)	0.32
Arg3500Gln (V9)	2p24	APOB	0.32
3:			
Pro12Ala (V10)	3p25	PPARG (MIM 601487)	0
A1166C (V11)	3q21-25	AGTR1 (MIM 106165)	0.32
4:			
Gly460Trp (V12)	4p16.3	ADD1 (MIM 102680)	0.32
G-455A (V13)	4q28	FGB (MIM 124830)	0.32
5:			
Arg16Gly (V14)	5q32-34	ADRB2 (MIM 109690)	0.32
Gln27Glu (V15)	5q32-34	ADRB2	0.32
G873A (V16)	5q23-31	ITGA2 (MIM 192974)	0.32
6:			
C93T (V17)	6q27	LPA (MIM 152200)	1.94
G121A (V18)	6q27	LPA	1.29
Thr26Asn (V19)	6p21.3	LTA (MIM 153440)	0
Thr26Asn (V24)	6p21.3	TNFb	0
G-376A (V20)	6p21.3	TNF (MIM 191160)	0.32
G-308A (V21)	6p21.3	TNF	1.62
G-244A (V22)	6p21.3	TNF	1.62
G-238A (V23)	6p21.3	TNF	0
7:			
Met55Leu (V25)	7q21.3	PON1 (MIM 168820)	0
Gln192Arg (V26)	7q21.3	PON1	0
Ser311Cys (V27)	7q21.3	PON2 (MIM 602447)	0.32
A-922G (V28)	7q36	NOS3 (MIM 163729)	0.32
C-690T (V29)	7q36	NOS3	0.32
Glu298Asp (V30)	7q36	NOS3	0.32
5G-6754G (V31)	7q21.3-22	PAI1 (MIM 173360)	0.32
G11053T (V32)	7q21.3-22	PAI1	0
8:			
Trp64Arg (V33)	8p12-11.2	ADRB3 (MIM 109691)	1.62
T-93G (V34)	8p22	LPL (MIM 238600)	2.59
Asp9Asn (V35)	8p22	LPL	0
Asn291Ser (V36)	8p22	LPL	0.32
Ser447Ter (V37)	8p22	LPL	0
9:			
Thr347Ser (V38)	11q23	APOA4 (MIM 107690)	0.65
Gln360His (V39)	11q23	APOA4	2.91
C-641A (V40)	11q23	APOC3 (MIM 107720)	9.06
C-482T (V41)	11q23	APOC3	0.65
T-455C (V42)	11q23	APOC3	0.32
C1100T (V43)	11q23	APOC3	1.29
C3175G (V44)	11q23	APOC3	0.32
T3206G (V45)	11q23	APOC3	0.32
5A(-1171)6A (V46)	11q23	MMP3 (MIM 185250)	0.32
G20210A (V47)	11p11-q12	F2 (MIM 176930)	0
10:			
Trp493Arg (V48)	12p13	SCNN1A (MIM 600228)	0.32
Ala663Thr (V49)	12p13	SCNN1A	0.65
C825T (V50)	12p13	GNB3 (MIM 139130)	0.32
11:			
-323 10-bp Del/Ins (V51)	13q34	F7 (MIM 227500)	0.32
Arg353Gln (V52)	13q34	F7	0.32
12:			
C-480T (V53)	15q21-23	LIPC (MIM 151670)	0
13:			
C-631A (V54)	16q21	CETP (MIM 118470)	0.32
C-629A (V55)	16q21	CETP	0.32
Ile405Val (V56)	16q21	CETP	0.32
Asp442Gly (V57)	16q21	CETP	0.32
Interon 14 G+1A (V58)	16q21	CETP	0
Intron 14(+3)Tins (V59)	16q21	CETP	0
Intron 1 TaqIB +/- (V60)	16q21	CETP	0
14:			
Intron 16 Ins/Del (V61)	17q23	ACE or DCP1 (MIM 106180)	6.5
Leu33Pro (V62)	17q21.32	ITGB3 (MIM 173470)	0.32
15:			
Cys112Arg (V63)	19q13.2	APOE (MIM 107741)	9.06
Arg158Cys (V64)	19q13.2	APOE	41.7
Ncol+/- (V65)	19p13.2	LDLR (MIM 606945)	0.97
Gly214Arg (V66)	19p13.3-13.21	ICAM1 (MIM 147840)	0
16:			
844 68bp-/Ins (V67)	21q22.3	CBS (MIM 236200)	0.32
Ile278Thr (V68)	21q22.3	CBS	0.32

* See .

### Phase 1: Detection of main individual effects of the polymorphisms

To evaluate the main effects of the polymorphisms on insulin resistance or T2DM, without taking into account the genotype for the other 67 polymorphisms, single locus logistic regression analyses were performed. Four polymorphisms (i.e., ACE, ADRB2, GNB3, APOC3) that provided significant results in these analyses were subsequently evaluated together in a multiple logistic regression model to determine of these effects were independent of one-another. We applied the procedure of Storey and Tibshinari [12] for controlling the overall error rate of multiple testing. In this procedure a statistical significance measure called false discovery rate (FDR) was defined, which is the expected proportion of false positives among the tests called significant [12,13].

### Phase 2: Detection of gene-gene interactions

To detect the haplotype interactions that influence the susceptibility to IGM and T2DM, we performed the following haplotype entropy procedure on every pair of SNP blocks and on every SNP pair.

Consider the unphased genotype data *G *= {*G*_1_,..., *G*_*n*_} on the *n *subjects at *m *loci from any two SNP blocks or two SNPs, where *G*_*i *_= (*G*_*i*1_,..., *G*_*im*_) and *G*_*ij *_takes values 0, 1, 2 according to whether its genetic haplotype at the locus *j *is homozygous with allele 0, or homozygous with allele 1, or heterozygous. Additional categories are created for missing two alleles (*G*_*ij *_= 9) and the presence of only one missing allele (*G*_*ij *_= 7 when allele 0 is missing at locus *j *and *G*_*ij *_= 8 when allele 1 is missing). Each *G*_*i *_can be partitioned into two sub-genotypes $G_{i}=(G_{i}^{\left(1\right)},G_{i}^{\left(2\right)}).$ We want to test the null hypothesis that the above two SNP blocks or two SNPs are independent. In the case of testing the independence between two SNPs, the problem can be viewed as testing the independence of two single SNP blocks with *m *= 2. Note that for *m *loci, the number of possible genotypes is 3^*m*^, much higher than 2^*m*^, the number of possible haplotypes. This means that the genotype space involved in modelling the dataset *G *has a much lower dimension than would be the corresponding haplotype space. Therefore, using haplotype frequencies to test the above null hypothesis would be more efficient than using the genotype-based *χ*^2 ^test and Fisher exact test in contingency tables. However, these haplotype frequencies are not directly available because the underlying haplotype pairs for these genotypes are unknown.

In an effort to tackle these limitations, Zhang et al. [8] proposed a haplotype-based test which takes into account both the haplotype-genotype relationship and unknown phases of these genotypes. It can be described as follows. Let *H *= {*H*_1_,..., *H*_*n*_} denote *n *candidate haplotype pairs that form the genotype dataset *G*, where *H*_*i *_= {*H*_*i*1_, *H*_*i*2_}, *H*_*is *_= (*H*_*is*1_,..., *H*_*ism*_),, *H*_*isj *_∈ {0,1}, *s *= 1,2. Given *G*, under the assumption of Hardy-Weinberg equilibrium, the likelihood *L*(*G|p*,*H*) is proportional to

$$\prod_{i=1}^{n}p(H_{i1})p(H_{i2})=\prod_{k=1}^{k_{0}}p{\left(H_{k}\right)}^{s_{k}},$$

as the function of unknown parameters $p=\left(p_{1},...,p_{m_{0}}\right)$ and *H*, where *H*_*k*_, 1 ≤ *k *≤ *k*_0 _are all the different haplotypes in *H*, $s_{1},...,s_{k_{0}}$ denote their respective frequencies, $p=\left(p_{1},...,p_{m_{0}}\right)$ denotes population frequencies of *m*_0 _possible haplotypes compatiable with *G*, and *p*(*H*_*i*1_) and *p*(*H*_*i*2_) are the population frequencies of haplotypes *H*_*i*1 _and *H*_*i*2_, respectively. Maximizing *L*(*G|p*, *H*) with respect to *p *under the contraints $\sum_{k}p_{k}=1,p_{k}\geq 0,1\leq k\leq k_{0}$, we obtain what is called profile likelihood $\prod_{k=1}^{k_{0}}{\left(\frac{s_{k}}{2n}\right)}^{s_{k}}$ for *H*. Taking the minus of the logarithm of this profile likelihood and dividing it by 2*n*, we obtain the entropy of the frequencies $s_{1},...,s_{k_{0}}$, namely

$$e(G|H)=-\sum_{k=1}^{k_{0}}\frac{s_{k}}{2n}\log\frac{s_{k}}{2n}.$$

Taking notice of the uncertainty of *H*, we define the haplotype-entropy *e*(*G*)of these genotypes as the minimal value of *e*(*G *| *H*) when *H *is running over all the possible sets of candidate-haplotype pairs. That is, *e*(*G*) is the value of *e*(*G *| *H*) when *H *attains the maximum of the above profile likelihood [9]. Zhang et al. [8] demonstrated that the smaller the haplotype-entropy, the stronger the interaction between the above two SNP blocks would be. This implies that when two SNP blocks are interacting, the haplotype entropy of their combined genotypes will be expected less than those generated from two independent blocks. Note that when the two SNP blocks are independent, exchanging the labels among sub-genotypes in one block would not change the distribution of *e*(*G*). To take advantage of this property in calculating the null distribution of *e*(*G*)(i.e., the distribution when the two blocks are independent), we conducted a permutation *π *only on the labels of sub-genotypes $G_{i}^{\left(1\right)},1\leq i\leq n$, which leads to a permutated genotype sample $G_{\pi }=\{(G_{\pi (i)}^{\left(1\right)},G_{i}^{\left(2\right)}),i=1,...,n\}$ with the haplotype-entropy *e*(*G_π_*) Repeating this permutation procedure say 200 times, we obtained a permutation distribution of *e*(*G*) as an approximation to the null distribution of *e*(*G*). The P-value of for an observed value of *e*(*G*) is then defined as the proportion of the times that *e*(*G*) is larger than *e*(*G_π_*). A Z-score can be also defined as in [8].

Our haplotype entropy procedure involves two stages: In the first stage the above permutation procedure is performed on each of three individual groups for interactions between and within haplotype blocks defined in Table 2. The glucose intolerance and T2DM predisposing interactions are found in the second stage by contrasting the interaction patterns observed for patients with the interaction patterns for controls. Thus, significant interaction between polymorphisms in one glucose intolerance state, but not in the others, implies is up- or down-interaction.

Here, the up-interaction means two blocks (or two polymorphisms) are independent in controls (NGT subjects) but become dependent blocks in cases (IGM or T2DM subjects). Similarly, we can define the down-interaction. The up-interactions would suggest that those interactions lead to a susceptibility to the disease, whereas the down-interactions could imply that the related interactions may have a protective effect on developing the disease [8].

Significant up- or down-interaction between two polymorphisms was established according to the criteria *P *≤ 0.05/*P *≥ 0.145). This was guided by a simulation study using a coalescent-based program called MS, by R Hudson [14]. We simulated genotypes for the three different situations (i.e., closely linked with a low mutation rate, weakly linked with a low mutation rate, and weakly linked with a high mutation rate) described by quantities (*θ*, *R*) = (4,4),(4,20), and (16,16). Here *θ *= 4*N*_*eμ*_,*R *= 4*N*_*e*_*r*, *N*_*e *_is the effective population size, *μ *is the total per-generation mutation rate across the region sequenced, and *r *is the genetic distance, in morgans, between loci. For each setting of (*θ*, *R*), we generated 20 samples of size 20 as control samples from a population in which the underlying two genotype blocks are independent, and 20 samples of size 20 as case samples from a population in which the two genotype blocks are dependent. We then applied our testing procedure to these data sets. The accuracy of our procedure can be measured by the quantities *F*_*a *_and *F*_*p*_, where *F*_*a *_is the proportion of false positives when the underlying two blocks in genotypes are independent, and *F*_*p *_is the proportion of successes in identifying an up- or down-interaction. Note that *F*_*a *_and *F*_*p *_can be roughly viewed as the type I error rate and the power of our testing procedure. For (*θ*, *R*) = (4,4),(4,20), and (16,16), (*F*_*a*_, *F*_*p*_) = (0.10,0.80),(0.05,0.80),(0.00,0.85) respectively. The results imply that if we use the thresholds *P *≤ 0.05/*P *≥ 0.145 in our multiple testing procedure, the overall type I error rate could be reasonably controlled at the level of 0.10 or less for the above simulated situations.

Unfortunately, since the above coalescent model may be biased against our data, the true type I error could be significantly different from 0.10. We have addressed this issue by correcting for multiple testing using the so-called Bayesian FDR-controlling procedure of Storey and Tibshirani [12].

The interactions that have been found in the above two stages may facilitate understanding of the pathological mechanisms involved in the diseases, as well as the further identification of some SNP blocks that provide significant association with the diseases only when their interactions with other blocks are taken into account. Note that the significant interactions between two polymorphisms detected in stage 1 of these analyses may be present irrespective of the insulin resistant state (i.e. is present in NGT, IGM and T2DM). These polymorphisms can be considered to be in complete association in this Caucasian study population. Such interactions cannot be directly used to discriminate between insulin resistant states (see also Table 4 and 5).

### Phase 3: Disease-predisposing allele combinations

The data obtained in Phase 2 of the study only provide information on which combinations of polymorphisms may be related to disease-risk, but this procedure does not identify risk-alleles. We have further analysed the gene-pairs identified in phase 2 to specify which combinations of alleles may actually be predisposing to disease. For this, frequencies of allele combinations in NGT, IGM and/or T2DM subjects were analysed using (comparisons of proportions; χ^2 ^tests).

Statistical analyses of phase 1 and 3 studies were done using SPSS 9.0 (Chicago IL, USA). The analyses in phase 2 were done using C and Splus 7.0 (Insightful Corp).

## Results

The general characteristics of the subjects (Table 1) show the expected metabolic differences between the NGT, IGM and T2DM subjects such as higher BMI, glucose, insulin, triglycerides and blood pressure and lower HDL (high density lipoprotein)-cholesterol in the T2DM patients. The groups did not differ with respect to gender and age. The NGT, IGM and T2DM groups were also comparable with respect to the presence of coronary heart disease.

### Phase 1: Main individual effects of polymorphisms

The results of logistic regression analyses (Table 3) suggest that the heterozygous genotype of ACE(ins/del) and ADRB2(gln27glu) and the homozygous genotype of the AA allele of APOC3 (C-641A) may predict disturbed glucose metabolism (i.e. subjects who were IGM or who had T2DM; P = 0.001, P = 0.002 and P = 0.008, respectively). A homozygous genotype for the minor allele of GNB3 (C825T), on the other hand, predicts protection from T2DM with a P-value that may be considered borderline significant (*P *= 0.0007). Applying the FDR-controlling procedure of Storey and Tibshirani [12] to these P-values in Table 3, we found that the expected proportion of false positives among our findings for ACE (ins/del), APOC3 (C -641A), GNB3(C825T) and apoC3 (C-641A) is 10%.

**Table 3:** Polymorphisms with main effects as independent predictors of disease status. q-value is the adjusted P-value using the procedure of Storey and Tibshirani [12].

	Frequency major/minor allele (%)^a^		NGT + IGM (0) vs T2DM (1)		NGT (o) vs IGM + T2DM (1)	
			
			Exp (B)	P-value (q-value)	Exp (B)	P-value (q-value)
ACE	54.2 I/39.6 D	II	1 (ref)	n.a.	1 (ref)	n.a.
(I/D)^b^		ID	1.6	0.14 (0.189)	4.0	0.001 ^c ^(0.006)
		DD	0.9	0.85 (0.535)	2.4	0.07 (0.110)
ADRB2	55.9 C/44.1	CC	1 (ref)	n.a.	1 (ref)	n.a.
(Gln27Glu)^b^	G	CG	1.8	0.042 (0.079)	3.9	0.002 ^c ^(0.007)
		GG	1.1	0.75 (0.506)	1.0	1.0 (0.591)
GNB3	72.3 C/27.7 T	CC	1 (ref)	n.a.	1 (ref)	n.a.
(C825T)^b^		CT	0.7	0.18 (0.189)	0.8	0.6 (0.473)
		TT	0.07	0.0007 ^c ^(0.006)	0.7	0.5 (0.430)
APOC3	45.9 C/45.3	CC	1 (ref)	n.a.	1 (ref)	n.a.
(C-641A)^b^	A	CA	0.6	0.16 (0.189)	1.1	0.7 (0.507)
		AA	1.4	0.32 (0.302)	4.1	0.008 (0.018)

^a^If total of minor + major alleles < 100%, this means that there were some missing genotype data;^b ^The 4 polymorphisms that had a P-value < 0.05 in individual logistic analyses were entered in this logistic regression analysis simultaneously, hence the effects of these polymorphisms are adjusted for one-another; ^c ^The expected proportion of false positives among these significant findings is 10% (FDR = 0.1).

**Table 4:** Haplotype Interactions *between *Blocks, i.e. interactions between polymorphisms in genes that are located on different chromosomes. Significant up- or down- interaction is indicated with bold typeface.

Block Pair/Chrom.Loc.Or SNP Pair/Gene Pair	NGT (control)		IGM		T2DM	
	
	P Value	Z Score	P Value	Z Score	P Value	Z Score
(1,5)/(1,5)	**0.025**	-2.11	**0.440**	-0.17	**0.145**	-1.00
(V2:V15)/(AGT:ADRB2)	0.005	-3.80	0.055	-1.73	<0.001	-5.62
(V4:V15)/(NPPA:ADRB2)	**0.015**	-3.00	**0.455**	0.11	**0.410**	-0.35
(V7:V16)/(SELE:ITGA2)	**0.900**	1.14	**0.365**	-0.78	**<0.001**	-0.44
(1,6)/(1,6)	**0.025**	-1.80	**0.860**	2.55	**0.925**	1.46
(V3:V21)/(NPPA:TNF)	0.060	-2.29	0.835	0.17	0.975	0.17
(2,3)/(2,3)	**0.010**	-2.45	**0.890**	1.13	**0.890**	1.17
(2,10)/(2,12)	**0.030**	-1.91	**0.585**	0.29	**0.200**	-0.66
(V8:V49)/(APOB:SCNN1A)	**0.010**	-3.12	**0.595**	0.20	**0.340**	-0.24
(V8:V50)/(APOB:GNB3)	**0.195**	-0.71	**0.245**	-0.82	**0.005**	-0.73
(2,13)/(2,16)	**0.240**	-0.61	**0.865**	1.15	**0.005**	-3.19
(V8:V56)/(APOB:CETP)	**0.760**	0.80	**0.915**	1.14	**0.005**	-0.70
(3,5)/(3,5)	**0.660**	0.38	**0.825**	0.97	**<0.001**	-2.72
(3,16)/(3,21)	**0.170**	-0.75	**0.410**	-0.15	**0.030**	-2.12
(5,16)/(5,21)	**0.615**	0.41	**0.910**	1.26	**0.025**	-2.35
(V15:V67)/(ADRB2:CBS)	**0.515**	0.33	**0.610**	0.17	**0.000**	-4.06
(6,10)/(6,12)	**<0.001**	-3.70	**0.755**	0.71	**0.915**	1.26
(V18:V50)/(LPA:GNB3)	**0.020**	-3.05	**0.905**	0.90	**0.935**	0.97
(V21:V48)/(TNF:SCNN1A)	**1.000**	0.75	**0.035**	-4.20	0.086	0.09
(6,11)/(6,13)	**0.205**	-0.58	**<0.001**	-3.45	**0.700**	0.59
(V18:V51)/(LPA:F7)	**0.695**	0.38	**0.010**	-2.44	**0.825**	0.50
(V18:V52)/(LPA:F7)	**0.370**	-0.21	**0.005**	-3.57	**0.900**	0.18
(6,16)/(6,21)	**<0.001**	-5.76	**0.335**	-0.34	**0.155**	-1.08
(V18:V67)/(LPA:CBS)	**<0.001**	-10.38	**0.670**	-0.20	**0.740**	-0.07
(7,12)/(7,15)	**0.205**	-0.81	**0.545**	0.07	**0.020**	-2.29
(V28:V53)/(NOS3:LIPC)	**0.570**	0.37	**0.215**	-0.94	**0.025**	-0.94
(V30:V53)/(NOS3:LIPC)	**0.045**	-1.96	**0.715**	0.56	0.105	-0.53
(8,14)/(8,17)	**0.415**	-0.13	**0.010**	-2.65	**0.190**	-0.88
(V34:V62)/(LPL:ITGB3)	**1.000**	0.66	**0.045**	-0.62	**0.895**	-0.14
(V37:V62)/(LPL:ITGB3)	**0.600**	-0.32	**0.890**	0.81	**0.050**	-0.30
(9,11)/(11,13)	**0.515**	0.04	**0.160**	-1.10	**0.030**	-1.99
(V39:V51)/(APOA4:F7)	**0.925**	-0.01	**0.575**	-0.58	**0.020**	-2.54
(V39:V52)/(APOA4:F7)	**0.760**	-0.53	**0.705**	-0.13	**0.030**	-2.38
(V41:V51)/(APOC3:F7)	**0.050**	-0.66	**0.460**	-0.32	**0.285**	-0.59
(V43:V51)/(APOC3:F7)	**0.005**	-1.43	**0.895**	1.14	**0.790**	0.68
(V43:V52)/(APOC3:F7)	0.055	-0.72	0.770	0.71	0.790	0.75
(12,13)/(15,16)	**0.005**	-2.54	**0.620**	0.07	**0.815**	0.96
(V53:V56)/(LIPC:CETP)	**0.025**	-2.55	**0.835**	-0.13	**0.585**	-0.06
(V53:V60)/(LIPC:CETP)	**0.040**	-2.13	**0.580**	0.21	0.100	-1.48
(13,15)/(16,19)	**0.575**	0.25	**0.665**	0.45	**0.005**	-2.51
(V56:V64)/(CETP:APOE)	**0.865**	0.85	**0.270**	-1.07	**0.020**	-2.90
(V54:V65)/(CETP:LDLR)	**0.215**	-0.84	**0.525**	0.42	**0.045**	-1.78
(V55:V65)/(CETP:LDLR)	**0.035**	-2.51	**0.820**	0.82	**0.255**	-0.90
(V56:V63)/(CETP:APOE)	**0.205**	-0.90	**0.030**	-2.84	**0.885**	0.85
(V60 :V65)/(CETP:LDLR)	**0.040**	-2.23	**0.935**	1.05	0.055	-1.87
(14,16)/(17,21)	**0.600**	0.27	**0.030**	-2.20	**0.600**	0.44
(V62:V67)/(ITGB3:CBS)	**0.185**	-0.59	**0.005**	-5.61	**0.695**	-0.28

**Table 5:** Haplotype Interactions *within *Blocks, i.e. interactions between polymorphisms that are located on one chromosome. Hence substantially more polymorphisms are in complete association than in Table 4. Significant up- or down interaction is indicated with bold typeface.

Block/Chrom.Loc. Or SNP Pair/Gene Pair	NGT (control)		IGM		T2DM	
	
	P Value	Z Score	P Value	Z Score	P Value	Z Score
1/1						
(V1:V4)/(MTHFR:NPPA)	0.005	-2.35	<0.001	-5.11	<0.001	-7.45
(V2:V4)/(AGT:NPPA)	0.705	0.16	0.095	-1.58	0.055	-2.12
5/5						
(V14:V15)/(ADRB2:ADRB2)	<0.001	-18.56	<0.001	-28.55	<0.001	-24.69
6/6						
(V19:V21)/(LTA:TNF)	<0.001	-2.38	<0.001	-20.07	<0.001	-23.57
(V20:V23)/(TNF:TNF)	0.005	-10.08	<0.001	-22.33	<0.001	-11.91
7/7						
(V31:V32)/(PAI1:PAI1)	<0.001	-9.16	<0.001	-17.77	<0.001	-27.53
8/8						
(V34:V35)/(LPL:LPL)	<0.001	-∞	<0.001	-25.74	<0.001	-24.56
9/11						
(V38:V40)/(APOA4:APOC3)	<0.001	-11.90	<0.001	-10.55	<0.001	-15.97
(V38:V41)/(APOA4:APOC3)	0.015	-2.700	<0.001	-1.49	<0.001	-6.33
(V38:V42)/(APOA4:APOC3)	<0.001	-10.50	<0.001	-4.48	<0.001	-23.90
(V38:V43)/(APOA4:APOC3)	**0.145**	-1.53	**0.025**	-3.00	**0.010**	-0.61
(V39:V40)/(APOA4:APOC3)	**0.405**	-0.69	**0.005**	-4.26	**<0.001**	-4.37
(V39:V42)/(APOA4:APOC3)	**0.715**	-0.34	**0.035**	-0.51	**0.050**	-0.33
(V40:V41)/(APOC3:APOC3)	<0.001	-12.81	<0.001	-10.77	<0.001	-21.46
(V40:V42)/(APOC3:APOC3)	<0.001	-11.49	<0.001	-7.39	<0.001	-50.76
(V40:V44)/(APOC3:APOC3)	0.015	-3.97	<0.001	-6.40	<0.001	-3.98
(V41:V42)/(APOC3:APOC3)	<0.001	-5.74	<0.001	-25.00	<0.001	-36.81
(V41:V43)/(APOC3:APOC3)	<0.001	-1.00	<0.001	-7.29	<0.001	-0.90
(V41:V44)/(APOC3:APOC3)	<0.001	-10.40	<0.001	-13.19	<0.001	-13.75
(V42:V43)/(APOC3:APOC3)	**0.800**	0.15	**<0.001**	-3.47	**0.925**	1.08
(V42:V44)/(APOC3:APOC3)	<0.001	-6.68	<0.001	-9.03	<0.001	-5.75
(V43:V44)/(APOC3:APOC3)	0.005	-5.92	<0.001	-16.54	<0.001	-7.07
10/12						
(V48:V49)/(SCNN1A:SCNN1A)	**0.585**	-0.84	**0.010**	-0.51	**0.045**	-0.76
11/13						
(V51:V52)/(F7:F7)	<0.001	-24.90	<0.001	-33.99	<0.001	-48.37
13/16						
(V54:V55)/(CETP:CETP)	0.005	-4.14	0.005	-4.78	<0.001	-0.73
(V54:V58)/(CETP:CETP)	**1.000**	1.00	**0.005**	-7.65	**1.000**	1.00
(V54:V60)/(CETP:CETP)	0.025	-2.64	0.015	-4.02	<0.001	-5.04
(V55:V56)/(CETP:CETP)	0.025	-2.30	<0.001	-4.71	<0.001	-8.17
(V55:V60)/(CETP:CETP)	<0.001	-21.23	<0.001	-43.59	<0.001	-55.84
(V56:V60)/(CETP:CETP)	0.050	-1.73	0.015	-2.68	<0.001	-5.72

Criteria for significant up- or down- interaction are *P *≤ 0.05 and *P *≥ 0.145. Thus, for instance, in the NGT subjects there is significant interaction between NPPA and ADBR2 (*P *= 0.015), which is absent in the IGM (*P *= 0.455) and the T2DM (*P *= 0.410; **Table 4**). Hence, IGM and T2DM status (i.e. a disturbed glucose metabolism) are associated with down-interaction between NPPA and ADRB2. When significant interactions between two polymorphisms is detected irrespective of the insulin resistant state (i.e. is present in NGT, IGM as well as in T2DM), this means that these polymorphisms are in complete association, for example, interaction between MTHFR and NPPA is *P *= 0.005 in the NGT subjects, *P *< 0.001 in the IGM subjects and *P *< 0.001 in the T2DM subjects (**Table 5**). This association is, by itself not informative for the insulin resistance state but may indicate involvement of the genes via a transitive interaction (see results section)

### Phase 2: Gene-gene interactions

We sought for pair-wise interactions between the 16 SNP blocks and between the polymorphisms. An up-interaction between two polymorphisms is claimed to be associated with disturbed glucose metabolism if significant evidence of the interaction between these two polymorphisms was found in the whole group of subjects with a disturbed glucose metabolism (those who were IGM or had T2DM) but not in the control (NGT) subjects. An up-interaction associated with T2DM is claimed if the interaction between these two polymorphisms was found significant in the T2DM subjects but not in the NGT or IGM subjects. Similarly we can define a down-interaction associated with disturbed glucose metabolism or associated with T2DM.

This revealed interacting pairs of genetic polymorphisms that are described in Table 6 (underlying data are in Table 4 and 5). Noteworthy, three of the four polymorphisms that were identified as potential main factors in the previous section, i.e. ADRB2(gln27glu), APOC3 (C-641A), GNB3(C825T), were also a member of one of these networks. If we accepted a FDR of 0.125, then 14 pairs of polymorphisms were detected to be associated with either IGM or with T2DM or with both (Table 6). This implies about 14×FDR = 1.75 pairs are expected to be false positive in these 14 pairs. Two important groups of networks can be identified. The first group, which might influence susceptibility to disturbed glucose metabolism (i.e. IGM and T2DM combined), was formed by down-interactions between LPA(G121A) and CBS(844 68bp-/Ins), between APOC3(C1100T) and F7(-323 10-bp Del/Ins), and between APOB(Thr7Ile) and SCNN1A(Ala663Thr) and up-interactions between APOA4(Gln360His) and APOC3(C-641A). The second group, which was found to be potentially associated with T2DM only, consisted of up-interactions between SELE(Leu554Phe) and ITGA2(G873A), between APOB(Thr71Ile) and GNB3(C825T), between APOB(Thr71Ile) and CETP(Ile405Val), between ADRB2(Gln27Glu) and CBS(84468bp-/Ins), and between APOA4(Thr347Ser) and APOC3(C1100T). In summary, at the FDR level of 0.125, polymorphisms in 7 genes (LPA, CBS, APOC3, F7, APOB, SCNN1A, APOA4) are involved in susceptibility to a disturbed glucose metabolism (subjects who were IGM or who had T2DM), and susceptibility polymorphisms in 9 genes (SELE, ITGA2, APOB, GNB3, CETP, ADRB2, CBS, APOA4, APOC3) may predispose to T2DM. Moreover, polymorphisms in 6 genes (ITGB3, CBS, LPA, F7, SCNN1A, APOC3) are involved in different networks that are IGM specific. If we relax the FDR level to 0.461 (in which P-values less than 0.05 are called significant), then 18 extra pairs of interacting polymorphisms can be identified (Table 6), but 32 × 0.461 = 14.75 of those pairs are expected to be false positive.

**Table 6:** Interactions between polymorphisms in genes that are associated with IGM or T2DM at the three FDR levels.

Block Pair/Chrom.Loc. Or SNP Pair/Gene Pair	FDR = 0.125		FDR = 0.461	
	
	IGM	T2DM	IGM	T2DM
(1,5)/(1,5)				
(V4:V15)/(NPPA:ADRB2)	--	--	Down	Down
(V7:V16)/(SELE:ITGA2)	--	Up	--	Up
(2,10)/(2,12)				
(V8:V49)/(APOB:SCNN1A)	Down	Down	Down	Down
(V8:V50)/(APOB:GNB3)	--	Up	--	Up
(2,13)/(2,16)				
(V8:V56)/(APOB:CETP)	--	Up	--	Up
(5,16)/(5,21)				
(V15:V67)/(ADRB2:CBS)	--	Up	--	Up
(6,10)/(6,12)				
(V18:V50)/(LPA:GNB3)	--	--	Down	Down
(V21:V48)/(TNF:SCNN1A)	--	--	Up	--
(6,11)/(6,13)				
(V18:V51)/(LPA:F7)	Up	--	Up	--
(V18:V52)/(LPA:F7)	Up	--	Up	--
(6,16)/(6,21)				
(V18:V67)/(LPA:CBS)	Down	Down	Down	Down
(7,12)/(7,15)				
(V28:V53)/(NOS3:LIPC)	--	--	--	Up
(V30:V53)/(NOS3:LIPC)	--	--	Down	--
(8,14)/(8,17)				
(V34:V62)/(LPL:ITGB3)	--	--	Up	--
(V37:V62)/(LPL:ITGB3)	--	--	--	Up
(9,11)/(11,13)				
(V39:V51)/(APOA4:F7)	--	--	--	Up
(V39:V52)/(APOA4:F7)	--	--	--	Up
(V41:V51)/(APOC3:F7)	--	--	Down	Down
(V43:V51)/(APOC3:F7)	Down	Down	Down	Down
(12,13)/(15,16)				
(V53:V56)/(LIPC:CETP)	--	--	Down	Down
(V53:V60)/(LIPC:CETP)	--	--	Down	--
(13,15)/(16,19)				
(V56:V64)/(CETP:APOE)	--	--	--	Up
(V54:V65)/(CETP:LDLR)	--	--	--	Up
(V55:V65)/(CETP:LDLR)	--	--	Down	Down
(V56:V63)/(CETP:APOE)	--	--	Up	--
(V60 :V65)/(CETP:LDLR)	--	--	Down	--
(14,16)/(17,21)				
(V62:V67)/(ITGB3:CBS)	Up	--	Up	--
(9,9)/(11,11)				
(V38, V43)/(APOA4:APOC3)	--	Up	Up	Up
(V39:V40)/(APOA4:APOC3)	Up	Up	Up	Up
(V39:V42)/(APOA4:APOC3)	--	--	Up	Up
(V42:V43)/(APOC3:APOC3)	Up	--	Up	--
(10,10)/(12,12)				
(V48:V49)/(SCNN1A:SCNN1A)	Up	--	Up	Up
# Pairs implicated in either IGM or T2DM or both (disturbed glucose metabolism)	14		32	

The indications Up and Down are associated with IGM if they are located in the IGM columns, and with T2DM if they are located in the T2DM columns. The FDR values indicate the expected proportion of false positives among the called significantly interacting pairs in the corresponding columns respectively.

In addition to these data on up- and down-interaction, we found that the following polymorphisms were in complete association with some members of the interaction networks (i.e., these polymorphism pairs are significantly interacting in the all three groups, NGT, IGM, T2DM): MTHFR(C677T), AGT(Met235Thr), LPL(Asp9Asn), F7(-323 10-bp Del/Ins), APOA4(Thr347Ser), APOC3(C-482T), APOC3(T-455C), APOC3(C3175), CETP(C-628A), CETP(Intron 1 TaqIB +/-) (see Table 5). This indicates that, besides the above-mentioned direct interactions via allele-coupling, additional polymorphisms may also, but perhaps indirectly, contribute to the interaction networks. For example, MTHFR is in complete association with NPPA (Table 5), AGT is in complete association with ADRB2 (Table 4), and NPPA is down-interacting with ADRB2 (Table 4). This implies MTHFR and AGT are transitively down-interacting with effects on susceptibility to a disturbed glucose metabolism.

### Phase 3: Disease-predisposing allele combinations

To substantiate the applicability of the data obtained in phase 2 of this study, we analysed the allelic distribution in the gene-pairs that were identified in phase 2. For the gene-pairs that were significantly associated with T2DM, ADRB2(Gln27Glu)-CBS(Ile278/Ins), APOB(Thr7Ile)-CETP(Ile 405Val), APOA4(Thr347Ser)-APOC3(C1100T), APOB(Thr7Ile)-GNB(C825T), ITGA2(G873A)-SELE(Leu554Phe), we found that when the C-allele of APOB was present, the distribution of the C and T alleles of GNB3 differed between T2DM (n = 207 of which 15.9% were T) and non T2DM (n = 222 of which 26.6% were T, P = 0.007). When the T allele of APOB was present, the distribution of the GNB3 alleles did not differ between T2DM (n = 106; 35.4% T) and non T2DM (n = 79; 47.2% T, n.s.). In other words, the allelic distribution of GNB3 between T2DM and non-T2DM is related to a specific genetic background for the APOB polymorphism, which implies gene-gene interaction. Likewise, we found that when the T-allele of SELE was present, the distribution of the A and G alleles of ITGA2 differed between T2DM (n = 12; 100% A) and non T2DM (n = 8; 62.5% A; P = 0.02). When the C-allele of SELE was present, the distribution of the ITGA2 alleles did not differ between T2DM and non T2DM. The gene-pairs that were significantly associated with IGM or T2DM subjects, APOB(Thr7Ile)-SCNN1A(Ale663Thr), F7(-323 10-bp Del/Ins)-APOC3(C1100T), APOA4(Gln360His)-APOC3(C-641A), LPA(G121A), CBS(Ile278/Ins) were analysed in a similar way. The distribution of the C and T alleles of APOC3 differed between disturbed glucose metabolism subjects (n = 71; 57.7% C) and NGT subjects (n = 16; 25.0% C, P = 0.018) when the insertion was present in F7, but not when the deletion was present. The distribution of the A and G alleles of LPA differed between subjects with a disturbed glucose metabolism (n = 68; 89.7% G,) and NGT (n = 12; 33.3% G, P < 0.001) when the insertion was present in CBS, but not when it was absent. The distribution of the C and A alleles of APOC3 differed between disturbed glucose metabolism (n = 422; 53.4% A) and NGT (n = 89; 36.0% A, P = 0.003) when the G allele of APOA4 was present, but not when the T allele was present. For the other gene-pairs we could not identify interaction using this approach.

## Discussion

The rationale behind the current study is that the effects of various risk genes on development of a complex disease will most likely not be independent from one-another. Therefore, such genes should be studied both individually and as members of genetic networks. T2DM is a well-known example of a complex disease and, in our view, the increased cardiovascular risk in T2DM implies that common metabolic pathways may be involved in development of T2DM and cardiovascular disease. We explored this possible involvement – including the possibility that gene-gene interactions are relevant for the effects of risk polymorphisms- in normoglycaemic, "pre-diabetic" (IGM) and diabetic (T2DM) subjects. Of note, the prevalence of coronary heart disease (CHD) was not different between the three disease groups. The involved risk genes and gene-gene interactions for subjects with a disturbed glucose metabolism (i.e. who were IGM or who had T2DM) or T2DM only, that we report here will therefore not result from imbalance in the distribution of coronary heart disease between the groups, but rather be related to their glucose tolerance status.

In phase 1 we performed single locus-based logistic regression analyses to identify main independent predictors for risk of a disturbed glucose metabolism or T2DM. This may be considered a more "traditional" approach. In phase 2 we used a novel approach to detect complex haplotype interactions (or allele coupling) among a set of polymorphisms to evaluate their effects on risk of glucose intolerance or T2DM. This resulted in identification of several networks of interacting genes. In phase 3 we assessed the applicability of data as obtained in phase 2 by constructing "disease predisposing allele combinations", thus showing that these add valuable information to that obtained in phase 1.

Twelve (LPA, CBS, APOC3, F7, APOB, SCNN1A, APOA4, SELE, ITGA2, GNB3, CETP, ADRB2) of the genes that we report here to be associated with susceptibility for a disturbed glucose metabolism and/or T2DM alone (when FDR = 0.125), actually reside on chromosomal regions that have previously been implicated in T2DM or related traits or have directly or indirectly been implicated in "diabetes associated" traits (for more detailed information see Tables 7 and 8). Our present findings may therefore be carefully considered to be duplications/confirmations of those previously published results. We propose that the fact that most of these genes/polymorphisms do not confer risk "on their own" but rather via interaction with other susceptibility genes may be one of the reasons for the apparent inconsistency in replication of the disease predisposing effects of several diabetes risk genes. In other words, the contribution of genetic polymorphisms to risk of human diseases with a complex genetic background such as T2DM may preferably be evaluated within their own genetic environment, i.e. while taking into account the genotype of other relevant polymorphisms carried by an individual. The approach described in phase 2 and 3 of this study provides a method for this.

**Table 7:** Known effects of the genes and polymorphisms involved in susceptibility for disturbed glucose metabolism (i.e. subjects who were IGM or who had T2DM, with the false discovery rate of 0.125), with focus on parameters that are directly related to an insulin resistance and T2DM.

*Gene*	*Gene or Chromosomal area was implicated in:*	*Variation*	*Polymorphism was implicated in:*
LPA 6q27	• The metabolic syndrome [15] which is characterised by insulin resistance	G121A	
CBS 21q22.3	• Hyperhomocysteinemia [16] which, in turn, is related to diabetic nephropathy [17].	Ile278Thr	• Homocysteinuria [18].
APOB 2p24		Thr71Ile	• Interaction between ApoB (Thr71Ile) and glucose tolerance on lipid parameters [19]
SCNN1A 12p13	• Same chromosomal region as GNB3	Thr663Ala	• SCNN1A(Thr663Ala) was associated with fasting insulin levels, even after correction for BMI [20].
APOC3 11q23	• Well-known diabetes region [21].	C-641A	
	• APOC3(C-482T) was associated with fasting insulin [22] and in interaction with LIPC -514 C>T also on glucose tolerance [23].		
APOA4 11q23	• Contained in the same region as Apo C-III	Gln360His	• Plasma glucose in women [24].
APOC3 11q23	• Well-known diabetes region [21].	C1100T	
	• APOC3(C-482T) was associated with fasting insulin [22] and in interaction with LIPC -514 C>T also on glucose tolerance [23]		
F7 13q34	• Chromosomal area implicated in the insulin-response-to-glucose in DM2 [25].	-323 10-bp Ins/Del	

**Table 8:** Known effects of the genes and polymorphisms involved in susceptibility for T2DM only (with the false discovery rate of 0.125), with focus on parameters that are directly related to insulin resistance and T2DM.

*Gene*	*Gene or Chromosomal area was implicated in:*	*Variation*	*Polymorphism was implicated in:*
ADRB2 5q32-34	• Susceptibility to DM2 [26], also in interaction with chromosome 10q23.3 [27].	Gln27Glu	• Independent contributor in the development of type 2 DM [28]
CBS 21q22.3	• Hyperhomocysteinemia [16] which, in turn, is related to diabetic nephropathy [17].	Ile278Thr	• Homocysteinuria [18].
APOA4 11q23	• Contained in the same region as Apo C-III	Thr347Ser	• Plasma glucose in women [24].
APOC3 11q23	• Well-known diabetes region [21].	C1100T	
	• APOC3(C-482T) was associated with fasting insulin [22] and in interaction with LIPC -514 C>T also on glucose tolerance [23]		
APOB 2p24		Thr71Ile	• Interaction between ApoB(Thr71Ile and glucose tolerance on lipid parameters [19]
GNB3 12p13	• Age-of-onset of DM2 [29].	C825T	• Implicated as independent contributor in development of DM2 [30]
			• Insulin resistance [31].
			• Insulin-mediated vasodilation [32].
APOB 2p24		Thr71Ile	• Interaction between ApoB(Thr71Ile and glucose tolerance on lipid parameters [19]
CETP 16q21	• CETP (Intron 1 TaqIB +/-) appeared to help significantly in identification of DM2 [33]	Ile405Val	
	• In our present study, CETP (Intron Taq1B+/-) was in LD with a.o. CETP(C-630A) and (Ile405Val).		
SELE 1q23-25	• Chromosomal area was implicated in the metabolic syndrome [34].	Leu554Phe	
ITGA2 5q23-31	• The ITGA2 C807T polymorphism may be associated with an increased risk of diabetic retinopathy [35]	G873A	• Risk factor for retinal vein occlusion [36].

For 2 of the 5 gene-pairs that were significantly associated with T2DM, we could show that the distribution of polymorphism A between T2DM and non-T2DM was dependent on which allele of polymorphism B was present. This indicates significant gene-gene interaction. Also for 3 of the 4 gene-pairs that were significantly associated with a disturbed glucose metabolism we could show gene-gene interaction. For the other gene-pairs we could not identify gene-gene interaction using this approach. One reason for this could be that the method used in phase 2 is more sensitive to networks that interact at a more complex level. Some of the gene-gene interactions may be more subtle than just pair-wise interactions, e.g. in predisposition to T2DM, there are pair-wise interactions between APOB(Thr7Ile) and CETP(Ile 405Val), but also between APOB(Thr7Ile) and GNB(C825T), so CETP and GNB3 polymorphisms may influence each-other via the APOB polymorphism or, alternatively, the effect of the APOB polymorphisms may be influenced by variations in both CETP and the GNB3.

Although the above results represent statistical association, the actual risk can hold true at the functional level. For instance, in our current analyses the gene-gene combinations that are involved in susceptibility for a disturbed glucose metabolism (Table 7) all include at least one gene involved in plasma lipid/lipoprotein metabolism. This suggests that disturbances in lipid metabolism may predispose to insulin resistance that is aggravated by the simultaneous presence of another polymorphism in a different gene. In the gene-gene combinations that predispose to T2DM (Table 8), this holds true for 3 out of the 5 combinations.

A comparison with previous data on type 1 diabetes (T1DM) that were obtained with a highly similar genotyping assay, unveiled that the gene-gene interaction network that was identified by Zhang et al. [8] as a potential genetic aetiology to T1DM, is different from the network for T2DM we present here. However, these networks do share a common set of polymorphisms including: NPPA(T2238C), APOB(Thr7Ile), ADRB2(Gln27Glu), NOS3(A-922G), APOA4(Gln360His), APOC3(C-641A), SCNN1A(Ala663Thr), LIPC(C-480T), LDLR(Ncol+/-), ACE (Intron 16 Ins/Del), and CBS(Ile278/Ins). This suggests that genes that predispose to T1DM may, especially in combination with other gene polymorphisms, add to the risk of T2DM and vice versa. This may relate to genes affecting beta cell function (e.g. NOS, ADRB2) and the effects of lipids on insulin secretion, the so-called lipotoxic effect on beta cells. (e.g. apoB, apoA4, apoC3, LIPC, LDL-R). These relations require further investigation.

The main limitation of the current study is the fact that, due to the small sample size, the epistatic interactions between SNPs must be quite strong to be detected. This also caused our selection of main effects of individual SNPs from the logistic regression to be only marginally significant. On other hand, although we have done some simulation study on the power of the haplotype entropy-based test, the issues on the power of haplotype entropy based SNP networking study and on an appropriate sample size for such a study have not been addressed. Despite of this limitation, we believe our study provides further evidence to support the hypothesis that the genetic basis of a disturbed glucose metabolism and T2DM is complex with many genes acting in concert in the transfer of risk.

## Conclusion

Our current approach to explore the presence of complex haplotype interactions (or allele coupling) among a set of polymorphisms has led to identification of several interacting networks of genes that may be relevant in predisposition to disturbed glucose metabolism or T2DM. Our current data thus imply that the combined presence of a specific set of risk polymorphisms, when simultaneously present, increases the risk of disease is indeed relevant in the transfer of risk. Such interacting polymorphisms may represent (functional) polymorphisms on different, physically independent genes. Simultaneous analysis of multiple risk polymorphisms located on different genes may be an important step in the identification of gene-gene interaction, and identification of the metabolic routes that are influenced by the "disease-predisposing allele combinations" may prove to be instrumental in further identification of the processes that underlie development of T2DM.

## Competing interests

MvG, JZ, CvdK, PS, EF: none to declare. TWAd.B. is currently employed by a company that manufactures and markets pharmaceuticals related to the treatment of diabetes and its complications.

## Authors' contributions

MvG and JZ contributed to interpretation of the data, performed the statistical analyses and drafted the manuscript; CvdK was involved in database management, quality control of the genotyping data, and critically reading and correcting the manuscript; PS supervised the genotyping assays; EF contributed to the design of the study, the interpretation of the data and commented on draft manuscripts; TWAdB contributed to the design of the CODAM study and commented on draft manuscripts;

All authors read an approved final version of the manuscript.

## Pre-publication history

The pre-publication history for this paper can be accessed here:



## References

[B1] O'Rahilly S, Barroso I, Wareham NJ (2005). Genetic factors in type 2 diabetes: The end of the beginning?. Science.

[B2] Mokdad A, Ford ES, Bowman BA, Dietz WH, Vinicor F, Bales VS, Marks JS (2003). Prevalence of obesity, diabetes, and obesity-related health risk factors. JAMA.

[B3] Baan CA, Poos MJJC Diabetes Mellitus. http://www.rivm.nl/vtv/object_document/o1259n17502.html.

[B4] Stern MP (2002). The search for type 2 diabetes susceptibility genes using whole-genome scans: an epidemiologist's perspective. Diabetes Metab Res Rev.

[B5] Putt W, Palmen J, Nicaud V, Tregouet DA, Tahri-Daizadeh N, Flavell DM, Humphries SE, Talmud PJ, EARSII group (2004). Variation in USF1 shows haplotype effects, gene: gene and gene: environment associations with glucose and lipid parameters in the European Atherosclerosis Research Study II. Hum Mol Genet.

[B6] Kubaszek A, Pihlajamäki J, Komarovski V, Lindi V, Lindström J, Eriksson J, Valle TT, Hämäläinen H, Ilanne-Parikka P, Keinänen-Kiukaanniemi S, Tuomilehto J, Uusitupa M, Laakso M, Finnish Diabetes Prevention Study (2003). Promoter polymorphisms of the TNF-alpha (G-308A) and IL-6 (C-174G) genes predict the conversion from impaired glucose tolerance to type 2 diabetes: the Finnish Diabetes Prevention Study. Diabetes.

[B7] Stumvoll M, Stefan N, Fritsche A, Madaus A, Tschritter O, Koch M, Machicao F, Häring H (2002). Interaction effect between common polymorphisms in PPARgamma2 (Pro12Ala) and insulin receptor substrate 1 (Gly972Arg) on insulin sensitivity. J Mol Med.

[B8] Zhang J, Liang F, Dassen WR, Doevendans PA, De Gunst M (2003). Search for haplotype interactions that influence susceptibility to type 1 diabetes, through use of unphased genotype data. Am J Hum Genet.

[B9] Zhang J, Vingron M, Hoehe MR (2005). Haplotype reconstruction for diploid populations. Hum Hered.

[B10] Cheng S, Grow MA, Pallaud C, Klitz W, Erlich HA, Visvikis S, Chen JJ, Pullinger CR, Malloy MJ, Siest G, Kane JP (1999). A multilocus genotyping assay for candidate markers of cardiovascular disease risk. Genome Res.

[B11] Kruijshoop M, Feskens EJ, Blaak EE, de Bruin TW (2004). Validation of capillary glucose measurements to detect glucose intolerance or type 2 diabetes mellitus in the general population. Clin Chim Acta.

[B12] Storey JD, Tibshirani R (2003). Statististical significance for genome-wide experiments. Proc Natl Acad Sci USA.

[B13] Benjamini Y, Hochberg Y (1995). Controlling the false discovery rate: a practical and powerful approach to multiple testing. Journal of the Royal Statistical Society B.

[B14] Hudson RR (2002). Generating samples under a Wright-Fisher neutral model of genetic variation. Bioinformatics.

[B15] Rainwater DL, Haffner SM (1998). Insulin and 2-hour glucose levels are inversely related to Lp(a) concentrations controlled for LPA genotype. Arterioscler Thromb Vasc Biol.

[B16] Aras O, Hanson NQ, Yang F, Tsai MY (2000). Influence of 699C-->T and 1080C-->T polymorphisms of the cystathionine beta-synthase gene on plasma homocysteine levels. Clin Genet.

[B17] Looker HC, Fagot-Campagna A, Gunter EW, Pfeiffer CM, Narayan KM, Knowler WC, Hanson RL (2003). Homocysteine as a risk factor for nephropathy and retinopathy in Type 2 diabetes. Diabetologia.

[B18] Kraus JP, Janosík M, Kozich V, Mandell R, Shih V, Sperandeo MP, Sebastio G, de Franchis R, Andria G, Kluijtmans LA, Blom H, Boers GH, Gordon RB, Kamoun P, Tsai MY, Kruger WD, Koch HG, Ohura T, Gaustadnes M (1999). Cystathionine beta-synthase mutations in homocystinuria. Hum Mutat.

[B19] Bentzen J, Poulsen P, Vaag A, Fenger M (2003). Further studies of the influence of apolipoprotein B alleles on glucose and lipid metabolism. Hum Biol.

[B20] Guo X, Cheng S, Taylor KD, Cui J, Hughes R, Quiñones MJ, Bulnes-Enriquez I, De la Rosa R, Aurea G, Yang H, Hsueh W, Rotter JI (2005). Hypertension genes are genetic markers for insulin sensitivity and resistance. Hypertension.

[B21] Baier L, Kovacs P, Wiedrich C, Cray K, Schemidt A, Shen GQ, Sutherland J, Thuillez P, Muller YL, Traurig M, Bogardus C (2002). Positional cloning of an obesity/diabetes susceptibility gene(s) on chromosome 11 in Pima Indians. Ann N Y Acad Sci.

[B22] Puigserver P, Spiegelman BM (2003). Peroxisome proliferator-activated receptor-gamma coactivator 1 alpha (PGC-1 alpha): transcriptional coactivator and metabolic regulator. Endocr Rev.

[B23] Jansen H, Waterworth DM, Nicaud V, Ehnholm C, EARS-II Study Group (2001). Interaction of the common apolipoprotein C-III (APOC3 -482C > T) and hepatic lipase (LIPC -514C > T) promoter variants affects glucose tolerance in young adults. European Atherosclerosis Research Study II (EARS-II). Ann Hum Genet.

[B24] Larson IA, Ordovas JM, Sun Z, Barnard, Lohrmann J, Feussner G, Lamon-Fava S, Schaefer EJ (2002). Effects of apolipoprotein A-IV genotype on glucose and plasma lipoprotein levels. Clin Genet.

[B25] Cai G, Cole SA, Freeland-Graves JH, MacCluer JW, Blangero J, Comuzzie AG (2004). Genome-wide scans reveal quantitative trait Loci on 8p and 13q related to insulin action and glucose metabolism: the San Antonio Family Heart Study. Diabetes.

[B26] Reynisdottir I, Thorleifsson G, Benediktsson R, Sigurdsson G, Emilsson V, Einarsdottir AS, Hjorleifsdottir EE, Orlygsdottir GT, Bjornsdottir GT, Saemundsdottir J, Halldorsson S, Hrafnkelsdottir S, Sigurjonsdottir SB, Steinsdottir S, Martin M, Kochan JP, Rhees BK, Grant SF, Frigge ML, Kong A, Gudnason V, Stefansson K, Gulcher JR (2003). Localization of a susceptibility gene for type 2 diabetes to chromosome 5q34-q35.2. Am J Hum Genet.

[B27] Wiltshire S, Hattersley AT, Hitman GA, Walker M, Levy JC, Sampson M, O'Rahilly S, Frayling TM, Bell JI, Lathrop GM, Bennett A, Dhillon R, Fletcher C, Groves CJ, Jones E, Prestwich P, Simecek N, Rao PV, Wishart M, Bottazzo GF, Foxon R, Howell S, Smedley D, Cardon LR, Menzel S, McCarthy MI (2001). A genomewide scan for loci predisposing to type 2 diabetes in a U.K. population (the Diabetes UK Warren 2 Repository): analysis of 573 pedigrees provides independent replication of a susceptibility locus on chromosome 1q. Am J Hum Genet.

[B28] Carlsson M, Orho-Melander M, Hedenbro J, Groop LC (2001). Common variants in the beta2-(Gln27Glu) and beta3-(Trp64Arg) – adrenoceptor genes are associated with elevated serum NEFA concentrations and type II diabetes. Diabetologia.

[B29] Wiltshire S, Frayling TM, Groves CJ, Levy JC, Hitman GA, Sampson M, Walker M, Menzel S, Hattersley AT, Cardon LR, McCarthy MI (2004). Evidence from a large U.K. family collection that genes influencing age of onset of type 2 diabetes map to chromosome 12p and to the MODY3/NIDDM2 locus on 12q24. Diabetes.

[B30] Kiani JG, Saeed M, Parvez SH, Frossard PM (2005). Association of G-protein beta-3 subunit gene (GNB3) T825 allele with Type II diabetes. Neuro Endocrinol Lett.

[B31] González-Núñez D, Giner V, Bragulat E, Coca A, de la Sierra A, Poch E (2002). Association of the G protein beta3 subunit T allele with insulin resistance in essential hypertension. Clin Exp Hypertens.

[B32] Mitchell A, Pace M, Nürnberger J, Wenzel RR, Siffert W, Philipp T, Schäfers RF (2005). Insulin-mediated venodilation is impaired in young, healthy carriers of the 825T allele of the G-protein beta3 subunit gene (GNB3). Clin Pharmacol Ther.

[B33] Relvas WG, Izar MC, Helfenstein T, Fonseca MI, Colovati M, Oliveira A, Ihara SS, Han SW, Las Casas AA, Fonseca FA (2005). Relationship between gene polymorphisms and prevalence of myocardial infarction among diabetic and non-diabetic subjects. Atherosclerosis.

[B34] Langefeld CD, Wagenknecht LE, Rotter JI, Williams AH, Hokanson JE, Saad MF, Bowden DW, Haffner S, Norris JM, Rich SS, Mitchell BD (2004). Insulin Resistance Atherosclerosis Study Family Study: Linkage of the metabolic syndrome to 1q23-q31 in Hispanic families: the Insulin Resistance Atherosclerosis Study Family Study. Diabetes.

[B35] Reiner AP, Agardh E, Teramura G, Gaur P, Gaur LK, Agardh CD (2003). Diabetes duration may modify the association between genetic variation in the glycoprotein Ia subunit of the platelet collagen receptor and risk of severe diabetic retinopathy: a working hypothesis. Thromb Haemost.

[B36] Dodson PM, Haynes J, Starczynski J, Farmer J, Shigdar S, Fegan G, Johnson RJ, Fegan C (2003). The platelet glycoprotein Ia/IIa gene polymorphism C807T/G873A: a novel risk factor for retinal vein occlusion. Eye.

